# Health Promotion Development in the Spa Treatment. Perspectives for the European Countries Learned from Poland's Experiences

**DOI:** 10.3389/fphar.2017.00029

**Published:** 2017-02-01

**Authors:** Joanna Woźniak-Holecka, Piotr Romaniuk, Tomasz Holecki, Aldona Frączkiewicz-Wronka, Sylwia Jaruga

**Affiliations:** ^1^Department of Health Promotion, School of Public Health in Bytom, Medical University of Silesia in KatowiceBytom, Poland; ^2^Department of Health Policy, School of Public Health in Bytom, Medical University of Silesia in KatowiceBytom, Poland; ^3^Department of Health Economics and Health Management, School of Public Health in Bytom, Medical University of Silesia in KatowiceBytom, Poland; ^4^Department of Public Management and Social Science, University of Economics in KatowiceKatowice, Poland

**Keywords:** health promotion, spa and wellness, spa services, health educators, health system and staff attributes

## Abstract

The main aim of the paper is to outline the perspective for future developments of the spa treatment in light of demographic transitions characterized by the increasing number of seniors, as well as changing expectations and health needs of younger population. We made a systematic review of literature referring to the experience of Poland, and similar experiences of other countries in Central Europe. Based on the existing knowledge we conclude that spa treatment should become one of the preferred directions of development of health systems in European countries. Moreover, we state that a desirable direction to modify the therapeutic paradigm used in spa treatment is to put a far-reaching greater emphasis on the provision of innovative health promotion, which is justified by both its effectiveness, and strongly good foundation for its provision in spas. For this purpose it is necessary to extend the specialized health sector personnel with qualified health educators, which will enable an effective implementation of health promotion actions and their proper alignment to the specific target groups. Developing this category of specialists will also enable other professionals to concentrate on therapeutic activity fitting their competence.

Spa treatment is perceived as an integral part of health care system in Poland and in other countries in Central and Eastern Europe. This is a result of historical conditions, that shaped the role of this form of therapy. Currently in Poland spa services annually are being used by more than 300,000 patients who receive treatment financed by public payers—most frequently the National Health Fund, but also the Social Insurance Institution or Agricultural Social Insurance Fund (patients undergoing treatment on the basis of a referral and the time delimitation). Additionally, there are about 100,000 commercial patients, including foreign ones, who pay out-of-pocket for the treatment, and do not require referral. Due to the increasing needs of the population arising of demographic phenomena the role of spa sector tends to even increase, and will probably demonstrate further increase of its potential as a response to the projected demographic and epidemiological changes in Poland and Europe (Holecki and Woźniak-Holecka, 2012).

The essence of the spa treatment is a 2- or 3-weeks long therapeutic, rehabilitative, and preventive procedure delivered in sanatoriums, spa hospitals, and spa outpatient clinics. It is offered primarily to patients suffering from chronic cardiovascular, orthopedic, rheumatological, and neurological diseases (Narodowy Fundusz Zdrowia, 2011). Regardless of the type of provider, every form of spa treatment includes provision of health promotion (health education, nutritional education, prevention of all levels, including rehabilitation; Ustawa z dnia 28 lipca, 2005; Rozporządzenie Ministra Zdrowia, 2015). Unfortunately, despite the fact, that health education is one of the basic services included in spa treatment, and as such, it should be a mandatory component of any treatment, it is considered now only as a supplement to the basic services. Meanwhile, the spa treatment based on practical tools of promoting health might, and should be, a crucial component of the continuum of medical therapy (Kalmus, 1998; Gutenbrunner et al., 2010).

## Anticipated trends in the development of spa services

The reason for the increase of needs regarding spa treatment are changes in the social structure, especially the increasing life expectancy contributing to the problem of aging populations in European countries (Jakovljevic and Laaser, 2015). In 2009, European Commission predicted increase in the proportion of people in retirement age in Europe to on average 30% in 2060, while in some countries the growth rate will be even higher—in Poland the share of the population above 65 years may be as high as 36% (European Commission, 2008). Undoubtedly, the Polish society is one of the fastest aging throughout the European Union (European Commission, 2015). While there has been a systematic increase in the average life expectancy of Poles by 6.1 years in women and 7.2 in men since 1990 (Główny Urząd Statystyczny, 2014; www.pwc.pl; 10 Trendów w Polskiej Ochronie Zdrowia, 2016), the 65+ population at the end of 2014 in nominal terms was nearly 5.9 million (Główny Urząd Statystyczny, 2014), and in the next 20 years, according to estimations, this number will increase to 8.5 million (www.pwc.pl; 10 Trendów w Polskiej Ochronie Zdrowia, 2016).

This obviously translates on morbidity and disability occurring in this population. Currently the number of older people suffering from chronic diseases in the age group 60–69 years is 60%, and in the group above 79 years—over 80% (Główny Urząd Statystyczny, 2015a,b). It is estimated that in the next 20 years the incidence of chronic diseases will increase by 2–8% (www.pwc.pl; 10 Trendów w Polskiej Ochronie Zdrowia, 2016). Regardless of the reason for this increase, whether it is a growing prevalence in this age group, or the greater share of seniors in the general population, it translates into a substantial increase in the number of patients requiring access to adequate health services. Polish seniors frequently suffer from cardiovascular diseases (hypertension and ischemic heart disease), lung diseases, osteoporosis, diabetes, visual impairment and hearing loss, arthritis, and cognitive disorders (Polsenior, 2012). Often there are several coexisting chronic diseases in one patient, which significantly increases the likelihood of organ damage limiting psychomotor performance.

In response to the upcoming demographic and epidemiological changes, as well as based on the latest concepts developed in health promotion, proactive measures should be intensified in these patient groups, who generate the highest health needs due to age or sex. Additionally, it is a fact that in spite of severe aging, modern Europeans tend to be more willing to keep active, while having more free time and often satisfactory income. Consequently, this leads to greater mobility manifested in higher propensity to travel abroad, including to the spas. This produces additional advantage and opportunity for applying intensified promotional activities for the elderly and chronically ill patients through the system of spa treatment (Braczkowski, 2014), where the highest rates of representativeness is present.

At the same time, as the experience of highly developed countries show, there is a second group of stakeholders consisting of young people, who pay high attention to a healthy lifestyle and physical fitness (Słomka and Kicińska, 2009). They are also potential consumers of spa-related health services, but require system of communication adequate to their profile and expectations, as well as structure and content of the offered service. This group expects possibilities of active leisure, nature touring, combined with a range of spa and wellness treatment, healthy diet, and physical activity (Górna, 2013). Finally, spa facilities located in attractive regions, but also possessing the infrastructure offering a rich set of procedures to improve the beauty and well-being, have favorable conditions to the implementation of professional trainings in nutritional education, health risk factors, or the general development of well-being through selected forms of physical activity.

Apart from clearly outlined two basic target group for spa services, there are also other target groups potentially interesting for the spa centers. These are groups which do not demonstrate clear symptom of illness, but reveal health needs which are often the consequence of negative effects of the civilizational development. There are also cases of occupation-related burden, such as stress, overstrain, burnout, or post-traumatic stress disorder (PTSD). Multistage spa therapy which is a combination of relaxation treatments with psychotherapy, health education, and prevention of somatic disorders may become an innovative health product and a good response for emerging new health needs of different population groups. The innovative nature of the proposed perspective fits both the specifics of the services to be provided in spas, in particular by strengthening the emphasis on promoting health and building a sustainable health potential of spa services beneficiaries, as well as in the increase of the scope and role of spa treatment in health care system. On the one hand, by expanding the target groups who are recipients of their offer, on the other one—by expansion of the spa sector potential in countries where historical tradition of its use cannot be matched to countries such as Poland and other countries of central Europe.

## Professional health educator as a component of spa services

Following the path of innovation coming from outside Europe, among the activities that should be adapted are modern tools of training a new categories of health professionals, since the expected state of things is that every spa facility employ a qualified health educators, e.g., as is the case of the American health care system (Lee et al., 2004). As shows the example of Poland, the role of health education is still underestimated in relation to the dominating paradigm of curative medicine, while application of education for patients is the responsibility of doctors and mid-level personnel, who in the face of an overload with other duties are not able to conduct it in a comprehensive and effective way.

These ideas fit into general challenges health care systems in the world are currently facing, like a sharp increase in spendings on health services, dynamic progress in medical technologies but also the growing social expectations regarding expensive ways of care for elderly (Borek-Wojciechowska and Kłokow, 2007). The wider competence of health providers in providing health promotion is a manifestation of these changes and an effort to address them. So should be also the shift in spa treatment. While in the past the responsibilities of spa sector were related only to a narrow area of rehabilitation and treatment of chronic diseases, now, in face of the new expectations and needs, it should demonstrate more focus on disease prevention, health education, and dietetics, aiming at expansion of patients' conscious learning capabilities, which should in turn result with health-related behavioral change.

A look at the case of spa sector in Poland gives an impression that there is a wide range of educational services related to disease risk factors, pharmacotherapy, and nutrition. Nonetheless, in reality the health education of patients in sanatoriums is highly imperfect and needs a strong systemic correction.

Due to its interdisciplinarity and clear links with such sciences as pedagogics or social psychology, health education presents itself as a useful tool not only in promoting health, but also in other areas of the health system, like prevention, rehabilitation or medical, and social care. To develop the profession of health educator should be therefore regarded as a contribution to the increase of quality and share of health promotion in system of health services, but also general raise of the efficiency of health system in producing better health outcomes. It is important, however, to design his role in a way, which should not be limited only to the transfer of knowledge about health determinants. In light of the latest research, such knowledge is not sufficient to change behavior and establish desired attitudes to health. This category of professionals should primarily get competence in building patient's health capacity and competence, which are the foundation for conscious behaviors (Woynarowska, 2016).

The transfer of service provision toward health education professionals will bring a range of tangible benefits. It will improve the quality of health education in spas, relieve doctors and nurses from carrying out time-consuming procedures related to health promotion and prevention, as well as will allow to better understand and meet patients' needs. In addition, lower cost of professionals' labor will bring savings in the health system and increase the availability of other services, as physicians released from a part of their current duties will be able to concentrate on treatment. The control of the quality and efficiency of health education services should include application of a separate pricing and financing procedures for these services (Rezner et al., 2013).

Innovative solutions in the field of health education may also contribute to building a competitive advantage, which is especially important in case of spa facilities located close to other institutions offering access to a similar scope of natural resources. To create innovative touristic products including effective health promotion, it is necessary to observe the changing trends in tourism, including health tourism. In this particular case, it is important to collect information about the quality, usability, and attractiveness of tourism products, as well as the specific needs of potential service receivers, in conjunction with the use of appropriate promotional channels (Dryglas, 2009).

Proposed trends in the development of spa services and related issue of health promotion can be cataloged using financial, managerial, and organizational factors. Unfortunately, one of the obstacles hardest to break are financial constraints (www.pwc.pl; 10 Trendów w Polskiej Ochronie Zdrowia, 2016) occurring in the form of public payer's limited budget, in conjunction with a diverse pricing of medical procedures and long waiting times for non-commercial spa treatment. These factors result in poor financial condition of companies providing spa services. Additionally, uncertainty associated with changes in legislation is also destructive for the development of the market. A potentially positive factor in turn, might be a clear increase in the phenomenon integrated into the concept of medical tourism, along with the high growth rate in demand for this category of services, estimated at 12–15%, in conjunction with international flows of patients: only in Poland there is almost 100,000 foreign patients every year. Moreover, the mere increase in the number of long-term services is ~6% per year (www.pwc.pl; 10 Trendów w Polskiej Ochronie Zdrowia, 2016). This expected change to occur in across the European Community should be used as an opportunity to take advantage of spa sector in the health care market.

## Conclusions

Application in the health system exclusively procedures of curative medicine is certainly insufficient. To meet the growing public expectations and health needs, the systems should move toward making the full use of all segments of the health care market, including a comprehensive spa treatment. This is all the more justified in light of the aging of population. As this is in part result of lengthening average life expectancy, to improve life quality of seniors becomes one of key priorities, along with limiting the effects of disability and preserving good mental health. For that reasons strengthening spa sector and applying wide range of health promotion activities on this basis appears to be an important element of the national health policy to limit the risk of disease. The aim should be to broaden the scope of holistically perceived services provided in spas, which should manifest itself in a continuity and complexity of the implemented actions aimed at strengthening the health potential of young people, and effectively supporting the elderly in the preventing social exclusion.

Although primarily addressed to seniors, spa health care in European countries should be opened also to the needs of young population struggling with health problems, or willing to increase its health potential, often in conjunction with health tourism. Activities aimed at this group of stakeholders should be treated as long-term socio-economic investment.

Moreover, because health promotion can be provided in spas in a most precise and comprehensive way, we should expect that in the near future it will change the nature of both spa services and spa tourism. Thus, it becomes a crucial link between tourism and spa treatment (Madeyski, 1998). The existing experiences may be quite a good foundation for coordination of tasks arising from the relationship of patients with physician, nurse, physiotherapist, and dietitian (Woźniak-Holecka, 2012). The fully effective coordinated system of spa service however should be based on professional health educators, whose additional advantage should be high efficiency provided at a relatively low cost.

## Author contributions

JW conceived the study, defined basic theses, and prepared draft of the paper; PR contributed to study design and final version of the paper; TH contributed to study design and final version of the paper; AF contributed to study design and final version of the paper; SJ did the literature review, prepared material for the paper, and contributed to paper draft.

### Conflict of interest statement

The authors declare that the research was conducted in the absence of any commercial or financial relationships that could be construed as a potential conflict of interest.
